# What happens next: The lingering effects of child abuse in a Mexican cohort

**DOI:** 10.1590/1516-3180.2024.0465.R1.21052026

**Published:** 2026-08-07

**Authors:** Efrén Romero Martínez, Mireya Robledo Aceves, Alejandro Barrón Balderas, América Aime Corona Gutiérrez, Eva Elizabet Camarena Pulido, Diego Magallón Picazo

**Affiliations:** I Pediatrics Department, Nuevo Hospital Civil de Guadalajara Dr Juan I Menchaca, Guadalajara, Jalisco, Mexico.; II Pediatrics Department, Toxicologic care and, Clinical Research Multidisciplinary Group, Nuevo Hospital Civil de Guadalajara Dr. Juan I Menchaca, Guadalajara, Jalisco, Mexico.; III Pediatrics Department, Toxicologic care and information Center, Health Science University Center, Universidad de Guadalajara, Guadalajara, Jalisco, Mexico.; IV Clinical Research Multidisciplinary Group, Health Science University Center, Universidad de Guadalajara, Guadalajara, Jalisco, Mexico.; V Clinical Research Multidisciplinary Group, Health Science University Center, Universidad de Guadalajara, Guadalajara, Jalisco, Mexico; VI Pediatrics Department, Nuevo Hospital Civil de Guadalajara Dr Juan I Menchaca, Guadalajara, Jalisco, Mexico.

**Keywords:** Child abuse, Cohort studies, Sports, Protective factors, Substance abuse, Mood disorders, Survivors

## Abstract

**BACKGROUND:**

Understanding the lingering effects of child abuse may improve healthcare pathways with preventive and supportive strategies.

**OBJECTIVE:**

To compare adverse outcomes between survivors of child abuse and non-exposed subjects.

**DESIGN AND SETTING:**

This is a cohort study conducted at the Pediatric Emergency Department of the Nuevo Hospital Civil de Guadalajara, Guadalajara, Mexico.

**METHODS:**

This cohort study of Mexicans compared adverse outcomes between survivors of child abuse and non-exposed individuals from 2011 to 2018. Additionally, we contacted survivors in 2020 to collect additional data, including sociodemographic and study variable-related information.

**RESULTS:**

This study included 300 survivors of child abuse, paired by age with 300 non-exposed subjects. Neglect and sexual abuse were the most frequent types of abuse. Among the survivors, there was a higher risk of hyperactivity with attention deficit [Relative Risk (RR) = 4.65(2.06–10.51); P = 0.0001], drug abuse [RR = 3.09(1.25–7.63); P = 0.0001], mood disorders [RR = 2.97(1.81–4.86); P = 0.0001], tobacco use [RR = 2.79(1.60–4.88); P = 0.0001], and engagement with substance abuse and sexual intercourse at a younger age. Non-exposed subjects had a higher level of education [RR = 0.65(0.49–0.87); P = 0.0001] and were more engaged in sports [RR = 0.31(0.18–0.52); P = 0.0001].

**CONCLUSION:**

Survivors of child abuse are at risk for psychological problems and substance abuse. Increasing sports engagement and school attendance may protect against adverse outcomes.

## INTRODUCTION

Child abuse refers to any type of maltreatment that causes physical, emotional, sexual, or negligent harm and, consequently, affects a child’s health, dignity, development, or survival.^1,2^ In addition to the traditional four types of child abuse (physical, emotional/psychological, sexual, and neglect), there are several other forms of maltreatment. These include peer-on-peer abuse (bullying), domestic violence, abandonment, harm to the unborn child (fetal abuse), Munchausen syndrome by proxy, ill-treatment of migrant and displaced children due to armed conflicts and violence, and children recruited as soldiers, hitmen, or assassin by drug cartels armed forces.^2,3^

In the United States, the prevalence of child abuse by a father or caregiver was nine per 1000 children.^4^ In Western Mexico, one hospital reported 318 cases per year.^2^ The real magnitude of child abuse is unknown, mainly because violence against children is often concealed. Family members are frequently involved, and survivors are afraid to speak out. Furthermore, in some regions, physical maltreatment is regarded as discipline. “Beating and scolding are the emblems of love” is an oriental proverb.^5^ Additionally, identifying child abuse without physical signs, such as psychological or emotional abuse, is challenging.^1^

The harm inflicted on a child has health and other consequences, including death. The World Health Organization estimates that deaths secondary to child abuse are more prevalent in children aged five years or younger, probably because this age group is entirely dependent on the caretaker–parent or family member. For children who survived child abuse, the consequences remain long after physical injuries have healed, negatively impacting neurologic development, emotional responses, cognition, and overall health.^1^ To provide comprehensive care and support, recognizing the long-term effects of child abuse is essential.

## OBJECTIVE

This study aimed to compare adverse outcomes between survivors of child abuse and non-exposed subjects.

## METHODS

This cohort study included patients aged < 16 years, suspected of child abuse, who were admitted to the Pediatric Emergency Department of the Nuevo Hospital Civil de Guadalajara from January 2011 to April 2018. Our Institution is a University Hospital with 3,500 to 4,000 pediatric emergencies a year. Patients were evaluated by a multidisciplinary group, the Programa de Atención Multiple al Maltrato Infantil (PAMMI, Comprehensive Care Program for Child Abuse), to determine if they were survivors of child abuse and, in confirmed cases, to provide care, including medical and legal aspects. The PAMMI database, first established in 2008, contains information on child abuse cases, including physical, emotional/psychological, sexual, negligent, abandonment, fetal, Munchausen syndrome by proxy, or bullying, and their follow-up.

In 2020, we conducted personal interviews with patients who were evaluated by PAMMI. The patients were divided into two groups: confirmed survivors of child abuse (exposed) and patients with maltreatment excluded (non-exposed), and compared. From both groups, we collected information concerning actual sociodemographic variables, psychological/emotional problems, school attendance and difficulties, nutritional profile, substance abuse, initial sexual experience, pregnancy, sports engagement, and rebellious behavior or delinquency. Exposed and non-exposed subjects were paired by age. Psychological/emotional problems were confirmed in the medical records. Prescribed medications were also documented.

As only some patients were. contactable in this cohort study, potential bias was considered. A sample size was calculated using a proportions formula for cohort studies, with a result of 150 exposed and 150 non-exposed. The number included in the study was doubled.

Statistical analysis is presented with means and standard deviation (SD) for continuous variables, with frequencies and percentages for categorical variables, and associations were determined using the Student’s t-test and chi-square test. Relative risk (RR) with 95% confidence intervals (95%CI) were calculated. Significance was set at P £ 0.05. We used logistic regression with the enter method and forward conditional selection for the final model to analyze independent risk factors. Sports engagement was considered a protective factor, and substance abuse, along with psychological/emotional problems, were risk outcomes. We present RR as crude RR (cRR) and logistic regression as adjusted RR (aRR).

The Nuevo Hospital Civil de Guadalajara Dr. Juan I Menchaca Research and Ethics Committee (CONBIOÉTICA-14-CEI-008-20161212) approved the study protocol (authorization and registration 00156) on August 18, 2020, and confirmed its compliance with the guiding framework of the Helsinki Declaration (1975, rev. 2013). Before the interviews, informed consent was obtained from patients aged > 16 years or from the parents or tutors in the case of younger patients. Patients aged ≥ 6 years also provided their written assent to participate.

## RESULTS

From January 2011 to April 2018, 3,238 survivors of child abuse were admitted to our Pediatric Emergency Department, which represented 11.7% of the 27,566 total admissions. Abused children ranged from one to 16 years of age, with a mean age of 11 years [± 4 years].

In 2020, we attempted to interview all survivors. However, we could only contact 300 [9.2%]. We also found that 16 [0.04%] patients were no longer alive, although their deaths were unrelated to child abuse. The main causes of death were cancer and related complications (e.g., leukemia, astrocytoma, neurofibrosarcoma, and sepsis), and chronic renal failure.

The age of the survivors at the time of the interview ranged from 4 to 25 years, with a mean of 17 years [± 4 years]. The most frequent types of abuse were negligence, sexual, and psychological. **Table 1** presents the types of abuse by age and sex of the 300 interviewed child abuse survivors admitted to our Pediatric Emergency Department between 2011 and 2018.

**Table 1:** Type of abuse by age and gender of the interviewed survivors (Guadalajara, 2011-2018)

Type of abuse	Female, n = 200 (66%) Age (years)				Male, n = 100 (34%) Age (years)				Total
	£ 2	3-5	6-12	13-16	£ 2	3-5	6-12	13-16	n (%)
Negligence	2	11	40	34			32	27	146 (48.5%)
Sexual	1	5	20	40		1	1	4	72 (24%)
Psychological			11	23			4	2	40 (14%)
Physical		1	4	6		4	8	2	25 (8%)
Abandonment					2	6			8 (2.6%)
Bullying			2				5		7 (2.3%)
Munchausen syndrome by proxy							2		2 (0.6%)
**Total**	3	17	77	103	2	11	52	35	300

School attendance was lower among survivors of child abuse. Consequently, a significant number of survivors were illiterate or had incomplete primary-level education compared to the non-exposed group, who generally had over nine years of education. School fights occurred more frequently among survivors of child abuse, and delinquency was found only within that group. Interestingly, nutritional status was not significantly different between the two groups (**Table 2**).

**Table 2:** Education, behavior and nutritional profile of survivors of child abuse versus non-exposed

Variable	Child abuse (n = 300)	Non-exposed (n = 300)	RR (95%CI)	P
**Education**				
Illiterate	38	1	2.09 (1.88-2.31)	0.0001
Primary incomplete	60	2	2.17 (1.95-2.41)	0.0001
Primary complete	16	1	1.93 (1.67-2.23)	0.0001
Secondary complete	19	8	1.43 (1.11-1.86)	0.03
High school complete	33	63	0.65 (0.49-0.87)	0.0001
**School fighting**	20	6	2.22 (1.09-4.4)	0.008
**Delinquency**	18	0	1.96 (1.72-2.23)	0.0001
Stealing	5	0		
Street fighting	13	0		
**Nutritional profile**				
Severe malnutrition	1	0	1.34 (0.60-2.98)	0.34
Mild malnutrition	30	15	1.37 (1.10-1.71)	0.02
Overweight	33	37	0.94 (0.72-1.22)	0.26
Morbid obesity	13	13	1.00 (0.68-1.48)	0.1

RR = relative risk; 95%CI = confidence interval at 95%;P = Chi square test statistically significant ≤ 0.05.

Sociodemographic variables and parental education were similar in both groups. The bivariate analysis did not reveal statistically significant differences. Therefore, these variables were not included in the multivariate model.

Attention-Deficit/Hyperactivity Disorder (ADHD) and mood disorders were significantly more prevalent in survivors of child abuse (P = 0.0001). Sadness, sleep disturbances, depression, and anxiety were some of the problems mentioned. Medical treatment with sertraline, risperidone, fluoxetine, olanzapine, alprazolam, or clonazepam was necessary for 41% [123] of the affected survivors. Recurrent substance abuse was also more likely among survivors of child abuse, especially drug use (P = 0.0001). Cannabis was the preferred psychoactive drug for both groups, alone or in combination with cocaine, inhalants, or amphetamines. Drug use also increased among the parents of the survivors (P = 0.0008). Sports engagement was less common in survivors of child abuse [P = 0.0001], while non-exposed subjects practiced soccer, gymnastics, cycling, basketball, and swimming for health and leisure, but not as high-performance athletes. The significance of these outcomes persisted after adjustment with logistic regression. (**Table 3**). The starting age for the risk outcomes (substance abuse, tobacco use, sexual intercourse, and pregnancy), defined in this study was younger among survivors, and a significantly larger number had offspring [69, 23% versus 40, 13%; P = 0.002] (**Table 4**).

**Table 3:** Logistic regression of risk variables among survivors of child abuse

Variable	cRR (95%IC)	P*	aRR (95%IC)	P**
ADHD	1.69 (1.42-2.02)	0.001	4.65 (2.06-10.51)	0.0001
Mood disorder	2.08 (1.52-2.86)	0.0001	2.97 (1.81-4.87)	0.0001
Tobacco use	2.10 (1.50-2.96)	0.0001	2.79 (1.60-4.88)	0.0001
Alcohol use	1.2 (0.98-1.58)	0.06	0.79 (0.45-1.41)	0.43
Drug use	4.05 (2.02-8.12)	0.0001	3.09 (1.25-7.63)	0.000.1
Drug use in parents	12.4 (1.8-85)	0.0001	15.7 (2.04-121.7)	0.008
Sports engagement	0.56 (0.49-0.65)	0.001	0.31 (0.18-0.52)	0.0001

ADHD = attention-deficit/hyperactivity disorder; cRR = crude relative risk; 95%IC = confidence interval of 95%

P* = statistically significant ≤ 0.05 according to the chi-square test; aRR = adjusted relative risk, logistic regression with the Hosmer-Lemeshow goodness-of-fit test and R^2^ (Cox-Snell, Nagelkerke)

P** = ≤ 0.05 according to the adjusted chi-square test.

**Table 4:** Start age for substance use, initial sexual experience and pregnancy

Start age (years)	Child abuse (n = 300)	Non-exposed (n = 300)	P*
Tobacco use	14 (7-22)	18 (16-20)	0.0001
Alcohol use	14 (7-18)	18 (15-20)	0.30
Drug use	13 (9-17)	18 (17-20)	0.0001
Initial sexual experience	14 (7-19)	17 (16-21)	0.0001
First pregnancy	16 (11-24)	19 (17-23)	0.0001

Results are presented in mean (range)

P* = Chi square test statistically significant ≤ 0.05.

## DISCUSSION

Approximately one in eight children admitted to our Pediatric Emergency Department between January 2011 and April 2018, were survivors of child abuse. This high frequency is reflective of referrals to the PAMMI group at our hospital.

The follow-up interviews revealed that Mexican survivors of child abuse have significantly lower educational levels and additional challenges, such as ADHD and school fighting. Child abuse impairs normal developmental processes, including cognitive, emotional, and social aspects. Maltreated children are at a higher risk of substandard academic achievement, decreased intelligence in childhood, and inferior educational levels. Notably, these risks may persist into adulthood.^6,7,8^

Child abuse affects memory, emotional regulation, and neurological connectivity of the inhibitory pathway. Similarly, these neurocognitive traits are compromised in ADHD. While child abuse and ADHD are significantly related, there is no consensus on the degree, type, or duration of maltreatment that could lead to ADHD.^6^

Other associations have also been identified. For example, milder forms of child abuse, such as inadequate infant attachment, have been related to school underachievement and mental health problems during adolescence.^9^ Emotional/psychological abuse and neglect have been associated with obesity in adulthood.^10^ Sexual abuse has been mostly linked to drug abuse during adolescence.^11^ Nevertheless, the extent of the neurological effects of child abuse during childhood and later in life has not been clearly determined.

Maltreatment survivors more often participate in violent acts or delinquency during adolescence and adulthood.^4,12,13^ We also found that stealing, street fighting, and school fights leading to expulsion or dropping out of school were only identified among the survivors. Commonly, abused children develop protective strategies that heighten their awareness of hostility. Moreover, survivors of abuse are vigilant of danger.^8^ These result from being hurt by the people who are supposed to care for them the most, often their parents and family.^4^ The constantly vigilant state could hinder stress management among the survivor, and increase the risk of recurrent violence.^8^

Young adults who have suffered child abuse are also at risk of mood disorders, such as suicidal behaviors, self-harm, depression, anxiety, and, to a lesser degree, bipolar disorder and psychosis. Therefore, they often require psychiatric or psychological services and medications, which over time could lead to the misuse or abuse of psychotropic drugs.^5,14^

Mills et al.^15^ found that Australian survivors of child abuse were at an increased risk of cannabis use during adolescence, which often continued into adulthood. Cannabis is the most commonly used drug worldwide. However, when cannabis is used before the age of 17 years, it is associated with alcohol consumption and smoking.^15^ In this study, early exposures (7-9 years of age) to drugs (e.g., cannabis, cocaine, amphetamines, alcohol, and tobacco) was demonstrated among the survivors of abuse. In contrast, non-exposed individuals had a start age of 15 to 17 years. In both groups, the start age for substance abuse was early, perhaps because, unlike in Australia, Mexico is a country involved in drug production and transit, which facilitates access for its residents.

Child abuse has also been associated with risky sexual behaviors, including early intercourse, transactional sex, multiple sexual partners, or unprotected sex, and with these, the associated risk of pregnancy and sexually transmitted diseases.^11,12,13^ In our study, the onset of intercourse and first pregnancy among survivors occurred during the adolescent years, starting as early as nine and 11 years, respectively, while in the non- exposed group, the earliest ages were 16 and 17 years. In Mexico, adolescent pregnancy is a public health problem with multifactorial causes, including gender inequity, social disparity, lack of education, early unions, and sexual abuse. Adolescent pregnancy in survivors of child sexual abuse is associated with a higher risk of miscarriage, postpartum depression, substance abuse, and unhealthy diets during pregnancy.^16^

Protective factors against the consequences of child maltreatment, including mood disorders, substance abuse, and aggressive behavior, have been explored. A high-quality relationship with a parental figure (e.g., mother or father) and school connection, along with neighborhood collective efficacy, are generally protective against offending behaviors. These factors could prevent unfavorable outcomes by fostering social engagement and supportive relationships with family, teachers, friends, and the community. We also found that non-abused subjects participated in sports significantly more than survivors. The bonds developed with their peers, along with involvement in a structured and supervised activity offer positive buffers. As Hirschi’s theory suggests, stronger social connections diminish the probability of antisocial behaviors.^4^

A comprehensive program that includes home visits, healthcare, and family support is essential for the prevention of child abuse.^17^ In Mexico, the Sistema Nacional para el Desarrollo Integral de la Familia (DIF, National System for Comprehensive Family Development) is the responsible institution for providing follow-up care to child abuse survivors. Unfortunately, available resources are insufficient to provide home visits, and the institution is not integrated with the healthcare system, which places child abuse survivors at a disadvantage. The social burden of this inadequate care includes a high prevalence of adolescent pregnancy, frequent school dropout, increased substance abuse, and involvement with drug cartels and organized crime. It is imperative to enhance the care pathways for child abuse survivors.

## CONCLUSION

Long-term consequences of child abuse include psychological issues, substance abuse, and at-risk adolescent sexual behaviors. Comprehensive programs that incorporate sports engagement and enhance school attendance could help mitigate these adverse outcomes in a cost-effective way.

## Data Availability

Data supporting the findings of this study are available upon request from the corresponding author, Mireya Robledo Aceves.
